# Distinct Roles of Dopamine and Noradrenaline in Incidental Memory

**DOI:** 10.1523/JNEUROSCI.0401-19.2019

**Published:** 2019-09-25

**Authors:** Tobias U. Hauser, Eran Eldar, Nina Purg, Michael Moutoussis, Raymond J. Dolan

**Affiliations:** ^1^Max Planck University College London Centre for Computational Psychiatry and Ageing Research, London WC1B 5EH, United Kingdom,; ^2^Wellcome Centre for Human Neuroimaging, University College London, London WC1N 3BG, United Kingdom, and; ^3^Departments of Psychology and Cognitive Sciences, Hebrew University of Jerusalem, Mount Scopus, Jerusalem 91905, Israel

## Abstract

Episodic memory is sensitive to the influence of neuromodulators, such as dopamine and noradrenaline. These influences are considered important in the expression of several known memory biases, though their specific role in memory remains unclear. Using pharmacological agents with relatively high selectivity for either dopamine (400 mg amisulpride) or noradrenaline (40 mg propranolol) we examined their specific contribution to incidental memory. In a double-blind placebo-controlled human study (30 females, 30 males in total), we show that a memory selectivity bias was insensitive to propranolol but sensitive to amisulpride, consistent with a dominant influence from dopamine. By contrast, a putative arousal-induced memory boosting effect was insensitive to amisulpride but was sensitive to propranolol, consistent with a dominant noradrenaline effect. Thus, our findings highlight specific functional roles for dopamine and noradrenaline neurotransmission in the expression of incidental memory.

**SIGNIFICANCE STATEMENT** Why some information is preferentially encoded into memory while other information is not is a central question in cognitive neuroscience. The neurotransmitters dopamine and noradrenaline are often assumed critical in influencing this selectivity, but their specific contributions remain obscure. In this double-blind, placebo-controlled, between-subjects drug study, we investigate the contributions of noradrenaline and dopamine to episodic memory. Using an incidental memory task, we find that blocking dopamine (400 mg amisulpride) eliminates a neural-gain related memory selectivity bias. Blocking noradrenaline function (40 mg propranolol), in contrast, abolishes an arousal-related memory enhancement. In this assessment of dopamine and noradrenaline neuromodulatory effects we reveal their specific contributions to episodic memory.

## Introduction

We encode many everyday experiences effortlessly into memory while others are subject to rapid forgetting. The determinants of what is stored, and what is lost, have been of interest to memory researchers for decades (McGaugh, 2000). The action of the neurotransmitters dopamine and noradrenaline are considered important in shaping whether, or not, an experience is consolidated as an enduring episodic memory trace (Strange et al., 2003; Shohamy and Adcock, 2010; Dunsmoor et al., 2015; Eldar et al., 2016b; Kempadoo et al., 2016; Takeuchi et al., 2016; de Quervain et al., 2017; Hämmerer et al., 2018).

Both dopamine and noradrenaline modulate hippocampal function, as well as that of other memory-related brain areas, via direct projections from ventral tegmental area (VTA)/substantia nigra (SN) and locus coeruleus respectively. A more complex picture is hinted at by recent reports which suggest that hippocampal dopamine arises not only from SN/VTA inputs, but also from locus coeruleus inputs, with the latter being critical for episodic memory (McNamara et al., 2014; Kempadoo et al., 2016; Takeuchi et al., 2016).

A key role for both dopamine and noradrenaline is to signal the relevance of an event, including its novelty, salience, or reward value (Strange et al., 2003; Shohamy and Adcock, 2010; Takeuchi et al., 2016; de Quervain et al., 2017). Experiences linked to such signals enhance subsequent memory performance. We previously showed that incidental memory can be boosted via emotional arousal, and this effect is influenced by noradrenaline (Strange et al., 2003). Others report similar effects that are dependent on the action of dopamine (Takeuchi et al., 2016).

**Figure 1.**
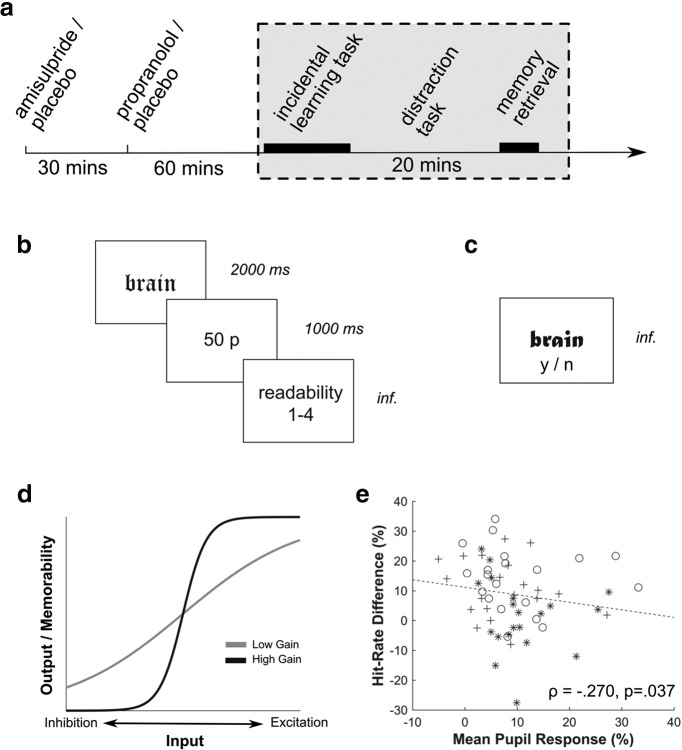
Neural gain during incidental episodic memory. ***a***, To assess specific effects of dopamine and noradrenaline, we administered either amisulpride or propranolol before an incidental learning task in a placebo-controlled design. Subjects were probed with a recognition task (***c***) ∼20 min after performing an incidental-learning task (***b***). ***b***, Incidental learning phase: subjects rated readability of common words, presented in two different fonts. Twenty-five percent of the words were randomly rewarded £0.50 to boost arousal (“50 p” or “00 p” feedback after word presentation). ***c***, Memory recognition test: subjects were asked to indicate whether a word has been shown during the first phase. Half of the words were presented in a different font compared with the original presentation (switch-font condition). ***d***, Predictions of neural gain. Neural gain is assumed to modulate how information is processed along neural populations. Under high neural gain (black), relevant features (such as the word shape in our experiment) are prioritized and their representation strengthened while unimportant features (here: word meaning) will be suppressed. Under low neural gain (gray), both relevant and negligible features are represented increasing the likelihood that both word shape and semantics will be stored in memory. ***e***, Pupil response indicates neural gain effects. Across all groups, we replicate our previous finding that pupil response during learning (as indirect indicator of neural gain) is linked to memory performance. Subjects with low pupil response (indicating high gain) show a stronger memory selectivity bias with a worse performance after a font switch (compared with a presentation in the same font; measured by hit rate). Subjects with larger phasic pupil response (indicating low gain) show less memory bias between same and switch-font condition. Shaded area in ***a***: time period of likely drug effect. inf, Unlimited response time; ○, placebo, +, propranolol, *, amisulpride.

One mechanism through which these neuromodulators might act is via an enhancement of neural gain (Servan-Schreiber et al., 1990; Aston-Jones and Cohen, 2005; Eldar et al., 2013). Neural gain characterizes how signals are processed and transformed within neurons and neural populations (Servan-Schreiber et al., 1990; Eldar et al., 2013, 2016b; Hauser et al., 2016; Mather et al., 2016). Under high neural gain, stronger input signals are enhanced and weaker inputs are suppressed (Fig. 1*d*). Under low neural gain all inputs are processed in a more egalitarian manner. Thus, a consequence of high neural gain is that salient signals alone prevail, whereas with low neural gain input stimuli are have a more holistic impact (Eldar et al., 2016a,b). Importantly, both dopamine and noradrenaline are known to modulate neural gain (Servan-Schreiber et al., 1990; Hauser et al., 2016).

Recently, neural gain has emerged as a mechanism of particular relevance to episodic memory formation (Eldar et al., 2016b). We previously demonstrated that subjects with high neural gain (inferred from pupillometry) preferentially encode stimulus dimensions critical for a cover task, while they ignore non-relevant stimulus features resulting in decreased recognition performance for such task-irrelevant stimulus dimensions (i.e., a memory selectivity bias). By contrast, subjects with low neural gain do not express any selectivity bias (Eldar et al., 2016b). In agreement with this, other studies show that arousal induction enhances memory for salient, goal-relevant, stimuli while impairing memory for other stimuli (Mather and Sutherland, 2011; Lee et al., 2015).

Here, in a memory task that probes recognition memory 20 min after an incidental word learning phase, we investigated the effects of catecholamine neuromodulation on neural gain and arousal. In a double-blind, placebo-controlled, between-subjects design we assessed the effects of drugs with relatively high affinity and specificity for either dopamine or noradrenaline. We found a double-dissociation evident in dopamine blockade eliminating a neural gain-related memory selectivity bias, while noradrenaline blockade attenuated an arousal-induced memory boost.

## Materials and Methods

### 

#### 

##### Experimental design and drugs.

We used a double-blind, placebo-controlled, between-subjects study design to assess the effects of dopamine and noradrenaline on incidental memory encoding. We selected agents with a high affinity and selectivity for either noradrenaline or dopamine. For noradrenaline blockade we used 40 mg of propranolol (β-adrenoceptor antagonist), a manipulation found previously to impact memory performance (Strange et al., 2003). For dopamine blockade we used 400 mg amisulpride (D2/D3 receptor antagonist), a dose known to impact on neurocognitive functioning (Kahnt et al., 2015; Kahnt and Tobler, 2017; Burke et al., 2018), opting for a D2/D3 receptor antagonist as there are no selective D1R antagonists available for human administration.

Because of distinct pharmacokinetics, and to conform with previously used drug protocols (Silver et al., 2004; Gibbs et al., 2007; De Martino et al., 2008; Kahnt et al., 2015; Hauser et al., 2017, 2018; Kahnt and Tobler, 2017), we administered these drugs at two separate time points (Fig. 1*a*). The amisulpride group received active drug 90 min before task onset, and a placebo 30 min after the first drug. The propranolol group first received placebo and subsequently the active drug. The placebo group received a placebo at both time points. The drugs were administered by a member of the research team (other than the experimenter), who was present while subjects imbibed the drugs.

To assess efficacy of pharmacological effects, we measured heart rate before drug administration and at task onset close in time to expected peak effect. We found that heart rate decreased in all groups (*F*_(1,57)_ = 221.06, *p* < 0.001), but the decrease was strongest in the propranolol group (time-by-drug interaction *F*_(2,57)_ = 4.18, *p* = 0.020; vs placebo: *t*_(38)_ = 2.57, *p* = 0.014; vs amisulpride: *t*_(38)_ = 2.37, *p* = 0.023), in line with expected physiological effects of propranolol (Koudas et al., 2009).

##### Subjects.

Sixty subjects were randomly assigned to one of three drug groups, assuring gender balance in all groups (10 females per group). Subjects were recruited from local subject pools and met the following inclusion criteria: absence of a history of neurological/psychiatric disorder, cardiac or other current health problems, medication use (except contraceptives), or known drug allergies. The groups were matched (Table 1) in terms of age, intellectual abilities (Wechsler, 1999), and mood at task onset (PANAS; Watson et al., 1988). Data from different tasks performed on the same subjects have been reported previously (Hauser et al., 2017, 2018). The study was approved by UCL research ethics and all subjects provided written informed consent.

**Table 1.** Characteristics of drug groups

	Placebo	Propranolol	Amisulpride	
Age	24.50 ± 4.16	23.15 ± 4.31	22.35 ± 2.21	*F*_(2,57)_ = 1.74, *p* = 0.185
IQ	112.45 ± 12.22	118.75 ± 8.55	114.60 ± 11.77	*F*_(2,57)_ = 1.70, *p* = 0.191
Positive affect	29.22 ± 10.47	27.15 ± 7.75	27.80 ± 8.12	*F*_(2,57)_ = 0.286, *p* = 0.752
Negative affect	11.45 ± 2.37	11.95 ± 4.87	11.25 ± 1.92	*F*_(2,57)_ = 0.236, *p* = 0.790

Values are mean ± SD. The drug groups did not differ in age, mood (PANAS), or intellectual abilities (WASI score based on subtests matrix reasoning and vocabulary).

##### Incidental memory task.

To probe incidental memory we adapted a task used in a previous study (Eldar et al., 2016b). This task design enabled us to assess two aspects of incidental memory encoding that we hypothesized would be affected by catecholamine functioning. First, we probed the role of both agents on putative neural gain-related memory effects, motivated by a previous finding that neural gain (as measured by pupil size) directly influences a selectivity in recognition performance when task-relevant features are altered (Eldar et al., 2016b). In an incidental learning phase, subjects were tasked to assess the readability of common words (details about the word stimuli cf. Eldar et al., 2016b) presented in uncommon fonts (Old English MT or Matura MT Script) on a scale of 1–4 (plus an additional key for unreadable words, which were subsequently excluded). Words were shown for 2000 ms and ratings were self-paced. We did not mention that subjects would later be probed on these words by means of a memory task. This entails that word semantics were irrelevant to the initial encoding task, and thus less likely to be processed under high gain (compare Fig. 1*d*; cf. Eldar et al., 2016b). We presented 104 words (medium to high frequency of 5 letter length, randomly assigned to condition) during a learning phase across four blocks, where the first and last four presented words of each block were discarded subsequently so as to avoid primacy/recency effects (cf. Eldar et al., 2016b).

Drug groups did not differ in how they performed this cover task. There was no difference in mean readability judgements (*F*_(2,57)_ = 1.51, *p* = 0.229), number of items labeled as non-readable (*F*_(2.57)_ = 0.51, *p* = 0.601), or reaction times for the readability judgment (*F*_(2,57)_ = 0.960, *p* = 0.389).

Following a 20 min break, during which subjects performed an unrelated perceptual metacognition task which had no memory component (random dots paradigm) or reward (Hauser et al., 2017), we conducted a memory recognition test (Fig. 1*c*) wherein subjects were asked whether they had seen the word in the first phase. The 72 originally presented words were complemented with 72 new words. Importantly, half of the original words were presented in a different font during the memory retrieval phase (switch font condition). This manipulation has been shown to substantially decrease performance for subjects with high, but not low, neural gain (Eldar et al., 2016b), because word semantics are only tangentially relevant to the original encoding task. The relatively short time between an incidental learning phase and a recognition test phase means that the drug treatments could affect both phases, rendering it challenging to apportion specific effects to either phase of the experiment.

A second aim was to assess an impact of catecholamine blockade on arousal-induced memory biases. To this end, we randomly rewarded 25% of all trials in the first encoding phase with £0.50. Reward was shown (for 1000 ms) immediately after stimulus presentation and before the readability rating (Fig. 1*b*). Subjects were instructed that this random lottery was entirely independent of their performance. To determine whether reward influenced subsequent episodic memory we used two distinct tasks. First, we assessed whether word recognition improved following receipt of reward. Second, we added a source memory task (Davachi et al., 2003; Gold et al., 2006; Kensinger and Schacter, 2006) in a final phase by presenting participants with two previously presented words and tasked them to select the word previously associated with reward (stimulus pairs consisted 1 rewarded and 1 unrewarded word).

To replicate a previously reported association between pupil response and font switching effects, we constructed the stimuli so that the foreground color (blue) was matched with the background (gray) in terms of luminosity. Moreover, we used a long intertrial interval (4000–6000 ms) between the word presentations during the initial learning phase to allow pupil size to return to baseline. After the memory recognition test, subjects performed two additional, unrelated tasks (modified exploration task: Wilson et al., 2014; and an information gathering task: Hauser et al., 2018).

##### Statistical analyses of behavior.

We assessed two distinct aspects that we hypothesized would be influenced by dopamine and noradrenaline: a font-switching induced memory selectivity biases, and an arousal-induced memory boosting by reward. To assess the first, neural gain-related hypothesis, we compared performance differences for words presented in the same versus a different font during a recognition memory task. For the font-switching analysis, we focused on non-rewarded stimuli so as to avoid confounding interactions from the reward manipulation.

We used repeated-measures ANOVAs to assess drug effects, and then used planned paired/independent-sample *t* tests to examine which drug differed from placebo (i.e., placebo vs propranolol, placebo vs amisulpride). Behavioral results are reported using Bonferroni correction for multiple comparisons.

To assess the effect of reward-induced memory biases, we compared word recognition performance (i.e., hit rates) between previously rewarded and unrewarded stimuli. In this analysis, we focused on stimuli that did not change font between training and testing phase so as to avoid potential confounds due to interactions with the font switching condition. In the source memory task, we assessed whether participants were able to correctly identify the previously rewarded word, and whether they performed above chance.

As our outcome measure, we focused on hit rates rather than signal detection theory-based measures, such as *d′*. We did so to ensure consistency with our previously reported analyses (Eldar et al., 2016b). Moreover, for several subjects *d′* was not computable for certain conditions, because performance was either at ceiling or floor (which renders the computation of *d′* prime impossible). However, when approximating *d′* using near-floor and near-ceiling substitute values, we found similar results as in our hit rate analyses. This suggests that the drugs act primarily on the sensitivity and not on a memory recognition bias.

##### Pupil analyses.

To examine a link between font switching and pupil response, we computed a metric of pupil responsivity for each subject as in our previous analysis (Eldar et al., 2016b). We used a EyeLink 1000 eye tracking device (SR Research) with a recording frequency of 1000 Hz. Triggers were sent using Psychtoolbox, and data were preprocessed and analyzed using FieldTrip (Oostenveld et al., 2011; cf. Allen et al., 2016). Based on the assumption that small pupillary responses indicate higher locus coeruleus/noradrenaline functioning (Aston-Jones and Cohen, 2005; Eldar et al., 2016b), we computed pupil response as the average peak of the stimulus-induced pupil dilation (1–4 s poststimulus onset) relative to baseline pupil size. To reach a similar sample size as previously, we pooled all subjects (see Results).

To assess whether our reward manipulation induced arousal, we further analyzed the outcome-evoked (reward vs non-reward) pupil responses between 0 and 4 s after outcome presentation. For both analyses, we linearly interpolated blinks and low-pass filtered the data (30 Hz). We then baseline-corrected the outcome-evoked responses using the 2 s before outcome onset and computed the difference in pupil response between the two conditions (reward − no reward). To assess significance, we applied a *p* < 0.05 cluster-based significance using permutation tests (height threshold *t* = 1.5, 500 permutations; cf. Hunt et al., 2013; Hauser et al., 2015).

## Results

### Pupil responses reflects gain-related memory selectivity bias

Neuroimaging and behavioral evidence suggests that pupil responses are useful indices of neural gain (Gilzenrat et al., 2010; Jepma and Nieuwenhuis, 2011; Eldar et al., 2013, 2016a,b; Warren et al., 2016). Before assessing the causal role of dopamine and noradrenaline we first replicated the previous finding that subjects with indices of high gain (smaller stimulus-evoked pupil responses during the learning phase) show a stronger memory selectivity effect (i.e., worse performance in the “switch font” condition) compared with subjects with indices of low gain (i.e., larger stimulus-evoked pupil response) using our previously established incidental memory paradigm (Eldar et al., 2016b). Specifically, we found a significant negative correlation (across all drug groups: ρ = −0.270, *p* = 0.037; Fig. 1*e*), such that subjects with a low pupil response (i.e., high neural gain) show a stronger font switching effect, thus replicating our previous findings (Eldar et al., 2016b).

There was no difference in pupil response between the groups (*F*_(2,59)_ = 1.06, *p* = 0.352; placebo: 10.1% ± 8.9; propranolol: 7.4% ± 7.6; amisulpride: 10.8% ± 7.0; placebo vs propranolol: *t*_(38)_ = 1.04, *p* = 0.305; placebo vs amisulpride: *t*_(38)_ = 0.29, *p* = 0.775; propranolol vs amisulpride: *t*_(38)_ = 1.49, *p* = 0.144). This is in line with a previous report that also did not find an effect of propranolol on pupil responses (Koudas et al., 2009). Correlations within each group were in the same direction as an overall group effect, but did not reach significance (possibly because of the smallish sample sizes; placebo: ρ = −0.191, *p* = 0.418; propranolol: ρ = −0.117, *p* = 0.624; amisulpride: ρ = −0.287, *p* = 0.219). These correlations did not differ between groups (placebo vs propranolol: *p* = 0.802; placebo vs amisulpride: *p* = 0.760; propranolol vs amisulpride: *p* = 0. 536, using permutation tests).

Last, a previous report found a decrease in mean pupil size after amisulpride administration (Samuels et al., 2006). To assess this, we averaged the pupil size across the entire trial and compared mean pupil diameter across drug groups. We found the amisulpride group had a smaller average pupil size compared with the propranolol and placebo groups (*F*_(2,57)_ = 5.591, *p* = 0.006; vs placebo: *t*_(38)_ = 1.79, *p* = 0.081, vs propranolol: *t*_(38)_ = 3.11, *p* = 0.004), replicating a previously reported effect of amisulpride.

### Dopamine blockade abolishes memory selectivity bias

**Figure 2.**
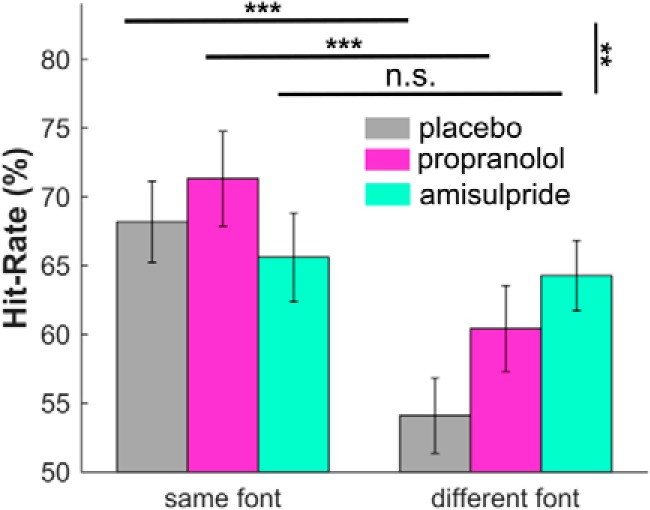
Blocking dopamine functioning reduces memory selectivity effect. Subjects generally show decreased recognition memory performance when words are probed in a different compared with the original font. However, this effect is only present in subjects under placebo and noradrenaline blockade (propranolol). Blocking of dopamine functioning (amisulpride) abolished the font switching effect, without impairing overall recognition performance. The findings indicate that this, neural gain-related memory selectivity bias is sensitive to dopamine but not noradrenaline function. Error bar: ± 1 S.E.M., ***p ≤ 0.001, **p < 0.01; n.s., *p* > 0.10.

To assess whether dopamine or noradrenaline influences a font-switch induced decrease in recognition performance (selectivity bias), we compared hit rate in both font conditions between the three drug groups. We found a consistent font-switch bias across all groups (repeated-measures ANOVA main effect of switch: *F*_(1,57)_ = 39.45, *p* < 0.001; Fig. 2), meaning that subjects performed generally worse when words were presented in a different font. However, this effect differed between drug groups (drug-by-font interaction: *F*_(2,57)_ = 7.54, *p* = 0.001, for non-rewarded trials alone; effect when including rewarded trials: *F*_(2,57)_ = 3.404, *p* = 0.040; same effects were found when using false alarms as covariate).

Subsequent planned comparisons showed the memory selectivity effect is present in the placebo (*t*_(19)_ = 6.01, *p* < 0.001) and propranolol groups (*t*_(19)_ = 5.03, *p* < 0.001), but is absent in the amisulpride group (*t*_(19)_ = 0.49, *p* = 0.630). Direct comparison confirmed that the memory selectivity effect is significantly less strong in the amisulpride than in placebo (*t*_(38)_ = 3.56, *p* = 0.002 corrected for multiple comparisons). We note that the drugs did not impact the general level of performance (main effect of group: *F*_(2,57)_ = 0.82, *p* = 0.447), or number of false alarms (*F*_(2,57)_ = 0.27, *p* = 0.763). There was no effect also on reaction times during the test phase (*F*_(2,27)_ = 1.06, *p* = 0.353). This means that blocking dopamine leads to a depletion of the selectivity bias in the absence of any impact on overall performance, suggesting that dopamine, but not (β-adrenoceptor related) noradrenaline has a causal influence on this gain-linked bias.

### Noradrenaline blockade reduces implicit arousal-induced memory boost

**Figure 3.**
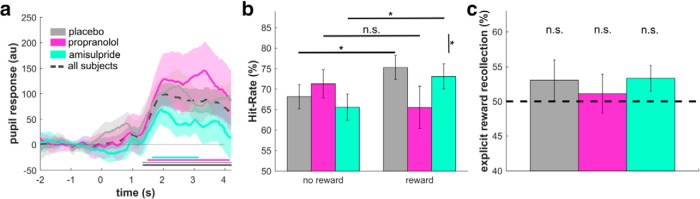
Implicit arousal-related memory boost eliminated by noradrenaline blockade. ***a***, Rare performance-independent rewards led to increased arousal as measured by a larger pupil dilation after rewarded (compared with non-rewarded) trials. The effect arose ∼2 s after reward presentation in all groups (horizontal lines: cluster-level significant group effects *p* < 0.05 using permutation tests). ***b***, The arousal-related rewards immediately following word presentation during incidental memory phase led to improved subsequent recognition. This effect was present both after placebo and dopamine blockade, but not after noradrenaline blockade. ***c***, The arousal-induced memory boost was not explicit. When subjects were asked to explicitly indicate which words were rewarded (source memory task), they did not perform above chance (dashed line) and the groups did not differ in their performance. Our findings suggest that the implicit arousal-induced memory boost primarily depends on β-adrenoceptor functioning. n.s., *p* > 0.05, *p < 0.05.

To investigate the role of dopamine and noradrenaline in an arousal-related boosting of episodic memory, we randomly rewarded 25% of all stimuli with £0.50 (Fig. 1*b*). Subjects were told that a random lottery determined whether each stimulus was rewarded and that these accumulated rewards would be added to subjects' reimbursement. We analyzed pupil dilation subsequent to reward presentation and found larger pupil dilation in all groups following reward compared with non-reward trials, 2–3 s after outcome onset (Fig. 3*a*). This supports an assumption that rewards modulated arousal (Allen et al., 2016).

We next investigated how this arousal manipulation influenced memory performance. We found enhanced recognition performance in some (Fig. 3*b*; reward-by-drug interaction: *F*_(2,57)_ = 4.41, *p* = 0.017, only same-font trials were analyzed; when including switch-font trials: *F*_(2,57)_ = 4.527, *p* = 0.015), but not all groups (main effect of reward: *F*_(1,57)_ = 2.02, *p* = 0.161; same effects were found when using false alarms as covariate). Subsequent analyses showed that words paired with a surprising reward had improved recognition performance in both placebo (*t*_(19)_ = 2.45, *p* = 0.024) and amisulpride groups (*t*_(19)_ = 2.19, *p* = 0.041). However, propranolol eliminated this arousal-related effect (*t*_(19)_ = −1.34, *p* = 0.197). This boosting effect of arousal on memory performance was significantly attenuated in the propranolol compared with placebo group (*t*_(38)_ = 2.48, *p* = 0.036 corrected for multiple comparisons). This means an arousal-induced memory recognition boost has a greater reliance on noradrenaline, but not D2/D3-related dopamine function.

Last, we assessed whether our reward manipulation also influenced subjects' episodic source memory. We thus used a source memory task (Davachi et al., 2003; Gold et al., 2006; Kensinger and Schacter, 2006) by presenting subjects with two previously presented words (1 rewarded, 1 unrewarded) and asked them to indicate which of the two were linked to receipt of reward. None of the groups performed above chance (Fig. 3*c*; placebo: *t*_(19)_ = 1.05, *p* = 0.308; propranolol: *t*_(19)_ = 0.395, *p* = 0.697; amisulpride: *t*_(19)_ = 1.79, *p* = 0.090), and the groups did not differ significantly from each other (*F*_(2,59)_ = 0.221, *p* = 0.802). This means that although rewards had a significant effect on memory recognition, subjects had no source memory for this effect.

## Discussion

The role of dopamine and noradrenaline as modulators of episodic memory has received much attention (Smith and Greene, 2012; Kempadoo et al., 2016; Takeuchi et al., 2016; McNamara and Dupret, 2017). Here, we show both neuromodulators influence episodic memory, and do so via distinct mechanisms.

We show that changing stimulus features, such as the font of a word, impairs word recognition 20 min after encoding and that the magnitude of this effect correlates with putative pupillometric indices of neural gain. However, this memory selectivity effect is abolished by manipulating dopamine function, but not noradrenaline function. This is of importance because pupil measures have traditionally been associated with noradrenaline rather than dopamine function (Joshi et al., 2016; Reimer et al., 2016; de Gee et al., 2017; Gelbard-Sagiv et al., 2018). Our results question this premise and point to a memory selectivity effect as preferentially dopamine driven. One way to interpret this result is to infer that neural gain is modulated not only by noradrenaline but also by dopamine. This has been proposed in for cognitive domains other than memory (Servan-Schreiber et al., 1990; Durstewitz and Seamans, 2008; Hauser et al., 2016), and is consistent with the observed effect of amisulpride on pupil diameter in our study. However, given existing uncertainties about the precise relationship between dopamine, pupil size, and neural gain, it remains possible that amisulpride exerts its effect in a non-neural-gain–dependent manner.

Our results chime with recent reports that propose the presence of catecholamine pluripotent neurons. These locus coeruleus neurons are considered to release not only noradrenaline but also dopamine, exerting an impact on hippocampal function during memory consolidation (Kempadoo et al., 2016; Takeuchi et al., 2016). This suggests locus coeruleus activity might mediate increased memory selectivity (alongside the altered pupil responses), via effects of released dopamine. Thus dopamine might serve as a priority enhancer to promote encoding of stimulus-relevant features, and attenuate encoding of peripheral stimulus-irrelevant dimensions.

A key finding was our observation that noradrenaline mediates an arousal-induced memory boost. Poststimulus presentation arousal, induced by a small, rare, reward led to improved subsequent recognition performance. This accords with previous studies demonstrating a memory boosting effect of arousing events, including that engendered by reward delivery (for review, see McGaugh, 2000). One possibility is that such a surprising event elicits a surprise prediction error in a frontoparietal network (Hauser et al., 2014) that in turn enhances stimulus encoding. However, our findings remain inconclusive as to whether this effect was driven by surprise (i.e., infrequent events) or by the rewarding nature of the stimulus, because reward delivery in our experiment is likely to elicit both a surprise prediction error and a reward prediction error. This question can be addressed in subsequent studies by use of non-rewarding rare stimuli, or by adding infrequent punishments.

We show that an arousal-induced performance boosting effect is specific to noradrenaline, and is insensitive to changes in dopamine D2/D3 functioning. The absence of an amisulpride effect is suggestive of an effect mediated via surprise, rather than a reward-related signal. This is in keeping with previous findings that reward-induced memory effects via long-term potentiation can be blocked by propranolol (Seidenbecher et al., 1997). Alternatively the memory effect of reward in our experiment might be driven by D1 primarily rather than by D2/D3 receptor activity, as is the case for other forms of memory (Müller et al., 1998).

Our findings emphasize caution against a strong inference on neurotransmitter function purely based on indirect measures alone, such as pupil response. We found no effect of propranolol on pupil response, in line with a previous report (Koudas et al., 2009). This suggests pupil responses might be primarily sensitive to α-adrenoceptor influences and less sensitive to β-adrenoceptor disruption (Koudas et al., 2009; Gelbard-Sagiv et al., 2018). We also did not find altered task-induced pupil responses after amisulpride, suggesting that a previous finding of increased light-induced pupil responses (Samuels et al., 2006) is distinct from an amisulpride effect on cognitive processes. However, in line with this previous report (Samuels et al., 2006), we found amisulpride influenced overall pupil size. Our results thus suggest that although propranolol and amisulpride modulate aspects of cognition, these effects can occur without directly affecting peripheral measures such as pupil response.

Multiple distinct processes contribute to the expression of episodic memory, and these processes are subject to the influence of different neuromodulatory systems. Our double-dissociation between noradrenaline and dopamine highlights the importance of targeted drug protocols that use drugs with a high specificity and allow a head-to-head comparison of different neurotransmitters. However, the current study design does not allow us to dissociate whether our drug manipulation primarily affected encoding or retrieval processes. Previous studies suggest that neurotransmitters, such as noradrenaline or cortisol might differently affect these phases (for review, see de Quervain et al., 2017). An extended time lap between encoding and retrieval would be needed to enable an apportioning of the specific drug effects to distinct phases. A further caveat is the unavailability of drugs that allow to specifically target D1 receptors in humans, which renders it difficult to examine the precise D1 contribution to higher-order memory processes.

In conclusion we show that both dopamine and noradrenaline contribute to incidental episodic memory, but have a different role altering specific memory biases. Our findings can thus help understand how potential pluripotent catecholamine neurons affect episodic memory in humans (Smith and Greene, 2012; Kempadoo et al., 2016; Takeuchi et al., 2016; McNamara and Dupret, 2017).
